# Synergistic Enhancement of Polymer–Cement Waterproof Coatings by Silane-Functionalized Cellulose Nanofibril

**DOI:** 10.3390/ma19081583

**Published:** 2026-04-15

**Authors:** Zizheng Wang, Kexin Xu, Xiaopeng Li, Qin Wang, Jian Wang, Sifan Zhao, Weidong Yang, Fanchao Zeng, Zhining Sun

**Affiliations:** 1 Institute of Advanced Materials, Beijing University of Civil Engineering and Architecture, Beijing 100044, China; 13804139001@163.com (Z.W.); 19819794548@163.com (K.X.); lixiaopengbucea@163.com (X.L.); 15201109348@163.com (J.W.); 18701252615@163.com (S.Z.); ywd202506@163.com (W.Y.); 2108590022116@stu.bucea.edu.cn (F.Z.); 2 Beijing Key Laboratory of Functional Materials for Building Structures and Environmental Remediation, School of Civil and Transportation Engineering, Beijing University of Civil Engineering and Architecture, Beijing 100044, China; 3 Ancient Bridge Research Institute, Beijing University of Civil Engineering and Architecture, Beijing 100044, China; 4 Shanxi Province High-Performance Admixture Materials Technology Innovation Center, Wanrong, Yuncheng 044200, China; 5 Beijing Yuandahongyu Waterproof Material Co., Ltd., Beijing 101116, China; leexl595@163.com

**Keywords:** modified cellulose nanofibers, styrene–acrylate, emulsion, mechanical properties, waterproof performance, durability

## Abstract

To enhance the mechanical properties and waterproof performance of polymer–cement (JS) waterproof coatings, cellulose nanofibrils (CNFs) were surface-modified using vinyltriethoxysilane (VTES). The modified cellulose nanofibrils (m-CNFs) were characterized by Fourier transform infrared spectroscopy (FTIR), scanning electron microscopy (SEM) analysis, and energy-dispersive X-ray spectroscopy (EDS). JS waterproof coatings incorporating m-CNFs were subsequently prepared. The performance and mechanism were systematically evaluated using the tensile strength, bonding strength, water absorption, contact angle, permeability test, durability test, scanning electron microscopy, Brunauer–Emmett–Teller (BET) and atomic force microscopy (AFM). The results indicated that the coating exhibited optimal performance when 1 wt% m-CNFs were incorporated. Under this condition, the tensile strength and bonding strength increased by 33.8% and 9.8%, respectively, while the 7-day water absorption decreased by 72.9%. The contact angle reached 97.1°, and the durability of the coating was also improved. Moreover, the amphiphilic nature introduced by silane modification effectively improved the interfacial adhesion between the organic and inorganic phases within the coating. In addition, due to their water absorption capacity, m-CNFs fill the micropores of the coating during the curing process and produce an internal curing effect, thereby reducing the porosity of the material. As a result of these synergistic effects, the mechanical strength and hydrophobicity of the JS waterproof coating are significantly enhanced. This study expands the application of CNFs, a sustainable nanomaterial, in building waterproofing materials.

## 1. Introduction

Studies have shown that approximately 80% of buildings experience leakage problems during their service life [1]. This phenomenon poses serious risks, often leading to structural damage, material deterioration, and potential threats to human life and property. Therefore, building leakage has become a critical issue that urgently needs to be addressed in the field of engineering.

JS waterproof coatings exhibit excellent waterproofing performance. After application, water in the coating decreases through two main processes: consumption during cement hydration and evaporation from the surface. This process promotes the gradual approach and coalescence of polymer particles in the polymer emulsion. As water continues to dissipate, further interactions occur between the polymer and the cement hydration products. Polymer chains become entangled around the hydration products, forming polymer films and generating a dense three-dimensional network. This structure effectively combines the elasticity of polymers with the strength and durability of cement, producing complementary properties [2,3,4]. Consequently, JS waterproof coatings have been widely applied in construction engineering. The polymer emulsion plays a key role in determining the waterproof and mechanical performance of the coating, influencing both its working mechanism and final waterproofing effectiveness. Among various polymer emulsions, styrene–acrylate (SA) emulsions have attracted considerable attention due to their strong adhesion and excellent film-forming ability [5,6]. However, SA emulsions still exhibit certain limitations, including insufficient tensile strength, poor water resistance, and limited compatibility with cement. In practical applications, the polymer phase often shows limited compatibility with inorganic hydration products, which can lead to weak interfacial bonding and structural defects. To overcome these drawbacks, researchers have made continuous efforts to improve the performance of these materials and have achieved significant progress. Nevertheless, the potential for further enhancement through the incorporation of nanomaterials remains to be explored [7].

Nanomaterials have been widely used in composite materials due to their remarkable surface effects, size effects, and quantum size effects. By filling internal defects within composites, nanomaterials can significantly improve material properties, including strength, surface roughness, and durability [8,9,10,11]. Among them, nano-silica has been extensively applied in polymer modification. Toughening can be achieved through selective debonding at the particle–matrix interface and plastic void expansion, while reinforcement occurs through the filling of micropores in the matrix. However, these mechanisms are often contradictory, making it difficult to simultaneously balance the toughness and strength of polymers [12,13]. Hajibabazadeh et al. [14] incorporated 5 wt% nano-silica into ethylene–propylene–diene monomer (EPDM), increasing the impact strength of the polymer material to 55 kJ/m^2^, while the elastic modulus decreased by 35%. Graphene, with its intrinsically high elastic modulus, can enhance the strength of materials while compensating for the loss of elastic modulus in polymers. However, its limited surface modifiability and high preparation cost restrict its practical application in polymer materials [15].

Cellulose nanofibrils, as a natural nanomaterial, possess abundant reserves, low density, biodegradability, high elastic modulus, and good surface modifiability, demonstrating great application potential [16]. Shankar et al. [17] incorporated CNFs into SA emulsions using in situ polymerization and ex situ mixing processes, with a maximum CNF concentration of 1.0 wt%. Compared with the pure copolymer, the resulting nanocomposite films exhibited improved mechanical properties, with a tensile strength of 7.8 MPa and an elongation at break of 1096%. In addition, the nanocomposites prepared by ex situ mixing showed a water contact angle of approximately 120°.

In recent years, nanocellulose has been increasingly incorporated into cement-based materials, demonstrating considerable application potential. Hu et al. [18] reported that, at an optimal dosage of 0.1–0.2%, cellulose nanocrystals (CNC) significantly enhance the mechanical properties of cement pastes with varying water-to-binder ratios. Specifically, the compressive strength, splitting tensile strength, and flexural strength were improved by approximately 18.8–22.14%, 19.4–26.38%, and 32–44.67%, respectively, indicating a pronounced reinforcing effect of CNC on cementitious materials. Furthermore, Zhu et al. [19] constructed an interpenetrating cellulose–cement dual-network structure, and the resulting cement-based fibrous fabric exhibited a nearly 20-fold increase in flexural toughness compared to conventional cement materials, along with excellent impact resistance and lightweight high-strength characteristics.

However, the application of natural cellulose nanofibrils is limited by its inherent hydrophilicity, which leads to poor dispersion in weakly polar matrices [20,21]. This is mainly attributed to the presence of numerous hydroxyl groups capable of forming hydrogen bonds, resulting in a strong tendency for self-aggregation [16]. Surface chemical modification is considered an effective strategy to overcome these limitations. Reactions between hydroxyl groups and organic reagents can improve the hydrophobicity of cellulose nanofibrils, thereby promoting better dispersion, which is a prerequisite for enhancing the mechanical properties of composites. Cabrera et al. [22] reported that silane coupling agents can undergo condensation reactions between their alkoxy groups and the hydroxyl groups on the surface of CNFs, forming stable chemical bonds. This process introduces organic functional groups onto the CNF surface and alters its surface polarity, transforming CNFs from strongly hydrophilic to moderately hydrophobic. As a result, the dispersion uniformity of CNFs in weakly polar matrices is improved, and the aggregation phenomenon is effectively suppressed. Dhali et al. [23] modified cellulose nanocrystals (CNCs) using VTES, and the water contact angle increased from 35° to 82°, confirming that the successful grafting of silane coupling agents with vinyl side groups enhanced the surface hydrophobicity.

The above studies demonstrate that CNFs can significantly improve the mechanical properties and impermeability of materials, indicating their considerable application potential. However, CNFs have not yet been applied in JS waterproof coatings, and the influence and mechanisms of CNFs in JS waterproof coatings remain unclear. In this study, CNFs were surface-modified using the silane coupling agent VTES. The modified cellulose nanofibrils was characterized using FTIR, BET, SEM and EDS analysis. Subsequently, m-CNFs were mixed with SA emulsion through an ex situ process and combined with cement and inert fillers to prepare waterproof coatings. The performance and mechanism were systematically evaluated through tensile strength, bonding strength, water absorption, contact angle, permeability test, durability test, and BET analysis. This study investigates the application of modified nanocellulose in JS waterproof coatings and suggests a potential strategy for mitigating building leakage issues, while providing useful insights into the use of CNF in organic–inorganic hybrid systems, particularly with respect to interfacial adhesion between the two phases.

## 2. Materials and Methods

### 2.1. Materials

In this study, P·I 42.5 Portland cement provided by China National Building Materials Group Co., Ltd. (Beijing, China) was used as the base cement material. The chemical composition of the cement was determined using X-ray fluorescence (XRF, PANalytical Axios, Almelo, The Netherlands) following standard analytical procedures, enabling the quantitative determination of the major oxide components.

The phase composition of the as-received cement was quantitatively analyzed without any hydration treatment. The cement powder was directly dried, ground, and sieved through a 75 μm sieve to ensure a uniform particle size for X-ray diffraction analysis. Quantitative phase analysis was performed using the internal standard method. Rutile (TiO_2_) was selected as the internal standard at a dosage of 10 wt%. The internal standard was thoroughly mixed with the cement sample and reground to ensure homogeneous dispersion. X-ray diffraction (XRD) measurements were conducted using an Ultima IV diffractometer (Rigaku, Tokyo, Japan) over a 2θ range of 5–80° at a scanning rate of 5°/min. The diffraction patterns were analyzed using the Rietveld refinement method to perform full-pattern fitting and quantitatively determine the crystalline mineral phases in the cement, including alite (C_3_S), belite (C_2_S), aluminate (C_3_A), and ferrite (C_4_AF). The reliability of the refinement was evaluated using the weighted profile R-factor (Rwp), and results with Rwp < 15% were considered acceptable. Preferred orientation effects, if present, were corrected using the March–Dollase model. It should be noted that the present analysis focuses on crystalline clinker phases in unhydrated cement. As a reference cement with negligible amorphous content was used in this study, no additional amorphous-phase quantification was performed.

The physical properties of the cement, including the setting time and compressive strength, were determined in accordance with the Chinese national standards GB/T 1346-2011 [24] (setting time) and GB/T 17671-2021 [25] (mortar strength), respectively. All tests were conducted under controlled laboratory conditions to ensure accuracy and repeatability. The detailed results are summarized in Table 1, Table 2 and Table 3.

Quartz sand and heavy calcium carbonate were employed as filler materials. The quartz sand possessed a particle size range of 80–120 mesh (125–180 μm), whereas the heavy calcium carbonate powder had a finer size of 300 mesh (48 μm).

**Figure 1:**
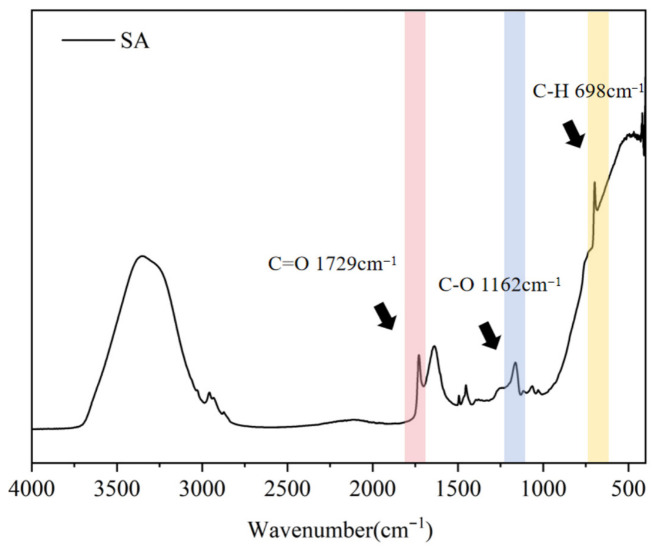
FTIR spectrum of styrene–acrylate emulsion.

The SA emulsion was prepared via copolymerization of styrene and acrylate, following previous work [4]. Its FTIR spectra were recorded at room temperature using a Fourier transform infrared spectrometer (Nicolet iS 10, Thermo Fisher Scientific, Madison, WI, USA). Spectra were collected in absorbance mode over the range of 400–4000 cm^−1^ with a resolution of 4 cm^−1^. Its key properties are listed in Table 4. The FTIR results (Figure 1) show characteristic peaks at 1700 cm^−1^ and 1150 cm^−1^, corresponding to C=O and C–O stretching vibrations, indicating the presence of carboxyl functional groups. The carboxylated cellulose nanofibril used in this study had a purity of 99%, with diameters ranging from 4 to 10 nm and a length of approximately 200 nm. They were purchased from Shanghai Macklin Biochemical Co., Ltd., Shanghai, China.

Other chemical reagents used in this study are listed in Table 5.

### 2.2. Fabrication of JS Waterproof Coatings

#### 2.2.1. Modification of CNFs

The silanization treatment of CNFs in this study was conducted with slight modifications based on the method reported by Frank et al. [26]. First, an ethanol–water solution with a mass ratio of 1.5:1 was prepared, and the pH was adjusted to approximately 3.5–4.0 using acetic acid. VTES (with a concentration of approximately 0.06 g/mL in ethanol) was then added to the ethanol solution and allowed to hydrolyze for 2 h.

Subsequently, the solution was transferred to a three-neck flask, and CNF powder (with a concentration of approximately 0.02 g/mL in ethanol) was added. The mixture was stirred at 60 °C and 300 r/min for 3.5 h. The suspension was then heated at 75 °C for 45 min to promote the grafting of VTES onto the surface of CNF particles. Afterward, the m-CNFs were cured at 110 °C for 45 min to facilitate further grafting of silanol groups.

The treated suspension was then redispersed in acetone and centrifuged twice at 2400× *g* for 10 min each to remove unreacted VTES. Finally, the m-CNFs were vacuum-dried at 60 °C to remove residual acetone. The dried product was ground into powder to obtain the final product, referred to as modified cellulose nanofibers.

#### 2.2.2. Reaction Mechanism of CNF Modification

**Figure 2:**
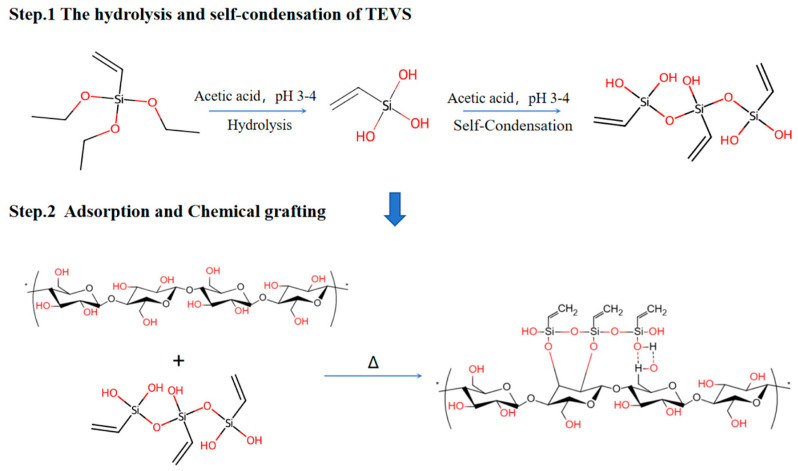
Reaction principle of CNFs’ modification The asterisk (*) indicates that the polymer chain continues with identical repeating units.

The modification of CNFs mainly involves the hydrolysis of VTES and its chemical reaction with the hydroxyl groups on the CNF surface. Under acidic conditions, the alkoxy groups (–OC_2_H_5_) in VTES are hydrolyzed into more reactive silanol groups (–Si–OH). This reaction is promoted by acid catalysis. The generated silanol groups exhibit strong hydrophilicity and enhanced chemical reactivity, enabling them to react with the abundant hydroxyl groups (–OH) on the CNF surface through chemical grafting to form stable siloxane bonds (–Si–O–C) [27,28]. The chemical reaction mechanism for the preparation of m-CNFs is schematically illustrated in Figure 2.

In addition, high-temperature curing further promotes condensation reactions between silanol groups as well as between silanol groups and hydroxyl groups on the CNF surface. This process increases the grafting density and improves the stability of the modified layer. Meanwhile, unreacted silanol groups undergo self-condensation to form a crosslinked siloxane network, which enhances the mechanical strength and hydrophobicity of the modified layer [29].

#### 2.2.3. Preparation of JS Waterproof Coatings

**Figure 3:**
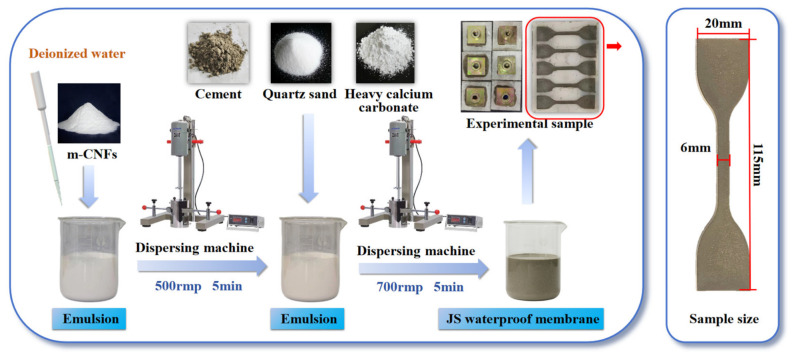
Preparation process of JS waterproof coating.

JS waterproof coatings were prepared according to the mix proportions listed in Table 6, and the preparation procedure is illustrated in Figure 3. m-CNFs or CNFs with contents of 0.5 wt%, 1 wt%, 1.5 wt%, and 2 wt% relative to the solid content of the SA emulsion were first dispersed in the SA emulsion as the polymer component. The sample without the addition of m-CNFs or CNFs was used as the control group and is denoted as P_0_C. The coatings containing m-CNFs were labeled as mP_x_C, while those containing CNFs were labeled as P_x_C.

**Figure 4:**
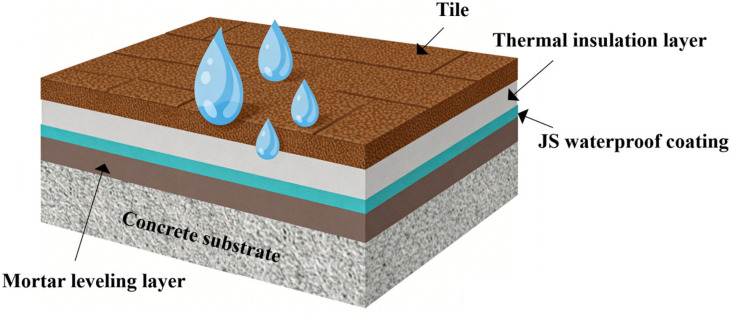
Schematic illustration of the coating system in a flooring structure.

A homogeneous JS waterproof coating mixture was prepared by combining SA emulsion, cement, quartz sand, and heavy calcium carbonate. The components were processed in a disperser at 700 r/min for 5 min to ensure adequate mixing. After mixing, the material was placed into dumbbell-shaped molds. The specimens had a total length of 115 mm, an overall width of 20 mm, a narrow section width of 6 mm, and a thickness of 1.5 mm. The molded samples were kept under curing conditions for 72 h. After curing for the required duration, the samples were removed from the molds based on the provisions of the Chinese standard GB/T 23445-2009 [30]. Figure 4 presents a schematic illustration of the coating system applied on a concrete substrate beneath the tile layer, representing its practical configuration in real engineering flooring systems.

### 2.3. Experimental Methods

#### 2.3.1. Mechanical Performance: Tensile and Bonding Strength

**Figure 5:**
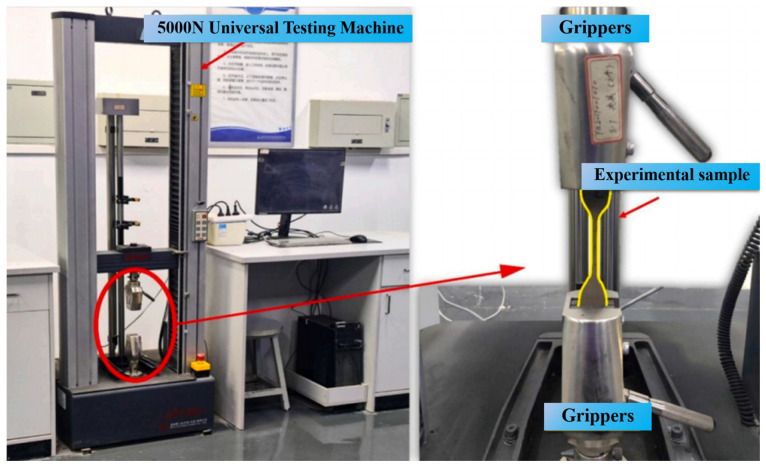
Tensile performance testing equipment.

The tensile behavior of the coatings was assessed in accordance with the Chinese standard GB/T 16777-2008 [31], which specifies testing procedures for building waterproof materials [4]. The specimens were fixed between the upper and lower grips of a universal testing machine (5000 N capacity, Figure 5). A continuous tensile load was applied to the specimens until rupture occurred. Based on the recorded data, the ultimate tensile strength (*T*) of the coating was determined based on Equation (1).

(1) $$T=\frac{F_{max}}{b\times d}$$

In Equation (1), *F_max_* refers to the maximum tensile load applied to the specimen (N), while *b* and *d* correspond to the specimen width (mm) and thickness (mm), respectively.

The adhesion properties of the waterproof coating were examined in accordance with the procedures outlined in the Chinese standard GB/T 16777-2008 for building waterproof coatings. For samples that did not undergo immersion treatment, bonding strength measurements were performed after curing for seven days. Prior to testing, an epoxy-based adhesive was applied to attach the loading fixture firmly onto the coating surface.

After the fixture was bonded, the coating material surrounding the upper fixture was carefully incised along the edge of the fixture using a sharp blade until the underlying substrate became visible. This preparation step created a square loading area measuring 40 mm × 40 mm. The prepared specimens were then positioned horizontally and maintained under standard laboratory conditions for 24 h, allowing sufficient curing of the adhesive and stabilization of the fixture–coating interface.

Subsequently, tensile loading was applied using a universal testing machine at a constant crosshead speed of 5 mm·min^−1^. The bonding strength reported in this study represents the average value calculated from five independently tested specimens.

#### 2.3.2. Water Absorption Test

The water absorption was tested according to the Chinese national standard GB/T 23445-2009, which describes methods for testing polymer–cement waterproof coatings. The prepared samples were cut into pieces of 20 mm × 20 mm. The pieces were immersed in water for 7 days. After that, the water absorption of the coating was calculated using Equation (2).

(2) $$W=\frac{m_{i}-m_{0}}{m_{0}}\times 100\%$$

In this expression, *m*_0_ refers to the initial mass of the specimen before exposure to water (g), while *m_i_* indicates the mass of the specimen after immersion (g).

#### 2.3.3. Water Permeability Test

**Figure 6:**
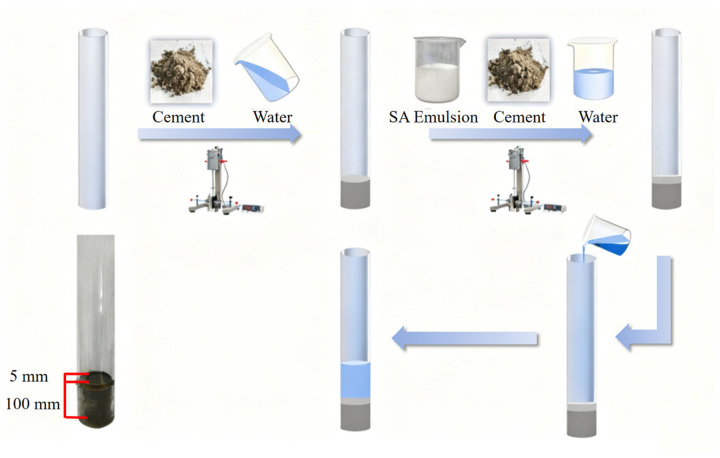
Schematic of the penetration resistance test.

The resistance of the JS waterproof coatings to water penetration was tested using an approach developed by our research team [4]. The general setup of the experiment is schematically presented in Figure 6. To produce the substrate, mortar was prepared using a water–cement ratio of 0.5 and a sand–cement ratio of 3:1. The freshly prepared mortar was poured into a cylindrical acrylic mold with an inner diameter of 50 mm, a wall thickness of 4 mm, and a height of 300 mm. During pouring, the height of the mortar column was kept constant at about 100 mm. The prepared mortar was cured for a period of seven days. After curing, the JS waterproof coating was prepared according to the previously specified proportions and then uniformly applied onto the exposed mortar surface. The coating thickness was kept constant at about 5 mm. After curing the coating for another seven days, tap water was poured into the acrylic tube until the water column height reached 100 mm. The water level variation in the acrylic tube was monitored for 1–3 days and was used as an indicator for evaluating the resistance of the coating to water permeation.

#### 2.3.4. Durability Test

The durability of the JS waterproof coatings was evaluated in accordance with the Chinese standard GB/T 16777-2008, which outlines the testing procedures for building waterproof coatings. Three different environmental conditions were applied to assess the stability of the coatings.

(1) High-temperature condition:

For the thermal resistance evaluation, the specimens were positioned horizontally on insulating supports and placed inside an electric blast drying oven. The temperature of the oven was maintained at (80 ± 2) °C, and the samples were subjected to this environment for 168 ± 1 h.

(2) Alkaline condition:

Under alkaline conditions, dumbbell-shaped specimens were submerged in a 0.1 wt% sodium hydroxide solution prepared with reagent-grade NaOH. The solution temperature was controlled at (23 ± 2) °C. Calcium hydroxide was gradually introduced until the solution reached a supersaturated state, ensuring that the liquid level remained at least 10 mm higher than the surface of the specimens. The samples were then stored in this environment for (168 ± 1) h. After immersion, each sample was removed, washed with clean water and subsequently dried. Subsequently, they were transferred to an oven maintained at (168 ± 1) h for 18 h and then allowed to cool naturally to ambient temperature.

(3) Ultraviolet (UV) exposure condition:

The resistance of the specimens to ultraviolet radiation was assessed by placing them in a horizontal position on glazed ceramic tiles. A thin layer of talcum powder was spread across the tile surface beforehand to prevent adhesion between the coating and the substrate. The prepared samples were then exposed inside a UV aging chamber for 720 h. After completion of the irradiation period, the specimens were removed and transferred to a desiccator to cool to standard room temperature prior to subsequent testing.

#### 2.3.5. Instrumental Analyses

The surface wettability of the coatings was evaluated by static water contact angle measurements using an optical contact angle analyzer (DSA100, KRÜSS GmbH, Hamburg, Germany). During the measurement process, a small water droplet was gently deposited onto the surface of the coating, and the corresponding images were recorded immediately after the droplet contacted the substrate. The captured images were subsequently processed using a five-point curve-fitting approach to determine the contact angle value. For each sample, five independent measurements were carried out at different positions, and the reported value represents the average of these measurements. The volume of the water droplet used in each test was controlled at 4 μL to ensure consistency.

The chemical structure of the CNFs was analyzed by Fourier transform infrared spectroscopy (FTIR). To verify the silane grafting of CNFs, the FTIR spectra of m-CNFs and unmodified CNFs were recorded at room temperature using a Fourier transform infrared spectrometer. Spectral acquisition was performed in absorbance mode within the wavenumber region of 400–4000 cm^−1^.

The specific surface area and pore structure of the samples were determined by Brunauer–Emmett–Teller (BET) analysis. The samples were first dried at 105 °C for 12 h, ground, and sieved through a 100-mesh sieve. Before testing, the samples were degassed in a vacuum degassing device at 150 °C for 6 h to remove adsorbed moisture and impure gases from the surface. The specific surface area and pore structure were analyzed using a surface area analyzer (ASAP 2460, Micromeritics, Norcross, GA, USA). High-purity nitrogen (99.999%) was used as the adsorption–desorption gas, and the measurements were conducted at liquid nitrogen temperature (77 K).

The microstructure and cross-sectional morphology of the coatings were characterized using scanning electron microscopy (SEM). The internal morphology and cross-sectional characteristics of the JS waterproof coatings were examined by means of a field-emission scanning electron microscope (ZEISS Sigma 360, Oberkochen, Germany). Prior to microscopic observation, the specimens were treated with a thin gold coating using a sputtering process for approximately 45 s at a current of 10 mA. This conductive layer was applied to enhance electron transport and suppress the surface charging effects during imaging. Following the coating step, the samples were secured on the microscope specimen holder for observation. Micrographs describing both the surface features and cross-sectional morphology were then obtained with an accelerating voltage of 3 kV.

The surface topography of the coatings was further analyzed using atomic force microscopy (AFM). Atomic force microscopy measurements were performed using single-crystal silicon wafers as supporting substrates. Prior to coating, the wafers were subjected to ultrasonic cleaning in absolute ethanol for 20 min to eliminate surface impurities. The cleaned substrates were then rinsed with deionized water and allowed to dry naturally in air. Subsequently, the wafers were briefly immersed in the coating solution and dried at room temperature. The coating thickness was controlled by adjusting the withdrawal speed during the dipping process, which ensured the formation of a uniform and smooth coating layer on the silicon substrate. To ensure moisture equilibration, the coated substrates were stored in a desiccator for 4 h before testing. The surface topography was finally analyzed using an atomic force microscope (Nanoscope IIIA, Bruker Dimension Edge, Ettlingen, Germany).

## 3. Results and Discussion

### 3.1. Modification of Cellulose Nanofibril

#### 3.1.1. Fourier Transform Infrared Spectroscopy Analysis

**Figure 7:**
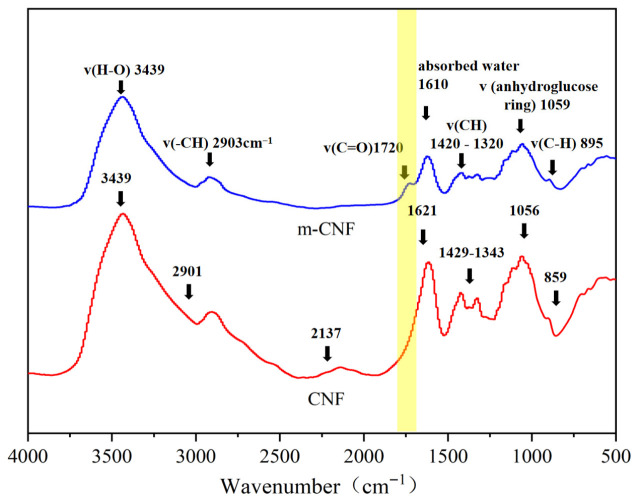
FTIR spectra of m-CNF and untreated CNF. The yellow shaded region highlights the characteristic peaks of m-CNFs.

The FTIR spectra of CNFs and m-CNFs are shown in Figure 7. The most prominent characteristic peak of CNFs appears at 3439 cm^−1^, which is attributed to the stretching vibration of intramolecular hydroxyl (O–H) groups. The absorption peak at 1621 cm^−1^ corresponds to the characteristic bending vibration of adsorbed water (O–H) [32]. The peaks between 1429 and 1343 cm^−1^ are associated with alkyl groups, including CH_2_ rocking vibration, symmetric bending, and C–H bending vibrations [33]. The absorption peak at 2901 cm^−1^ originates from the C–H stretching vibration of aliphatic CH_2_ groups, while the peak at 1059 cm^−1^ is related to the C–O–C bond in the glycosidic linkage.

Compared with unmodified CNFs, m-CNFs exhibit a new absorption peak at 1680 cm^−1^, which corresponds to the C=O stretching vibration of ester groups. This is attributed to the esterification reaction between the carboxyl groups (–COOH) on the surface of the carboxylated cellulose nanofibrils used in this study and the hydroxyl groups (–OH) of silanol, which to some extent confirms the successful grafting of VTES.

However, the characteristic absorption peaks of siloxane bonds (Si–O) and silicon–carbon bonds (C–Si), typically located at 1000–1200 cm^−1^ and 800–1000 cm^−1^, respectively, were not clearly observed in the results. This may be due to the overlap of these peaks with the C–H absorption peaks of alkyl groups, which makes the target peaks difficult to distinguish. Therefore, additional characterization methods are required to further verify the successful grafting of the silane coupling agent.

#### 3.1.2. Microscopic Observation of CNFs and m-CNFs

**Figure 8:**
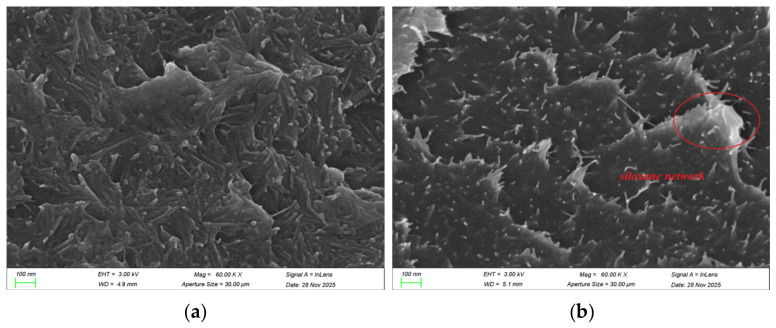
SEM image of CNFs and m-CNFs: (**a**) SEM image of CNFs, (**b**) SEM image of m-CNFs.

The morphology of CNFs and m-CNFs was observed using scanning electron microscopy, as shown in Figure 8. The original CNFs exhibit a flexible filamentous structure, interwoven to form a three-dimensional network morphology, with a typical length of approximately 200 nm and a width of about 4–10 nm. After silane modification, a noticeable thickening of the cellulose chains can be observed (Figure 8b).

This phenomenon is mainly attributed to the gradual growth of a polysiloxane layer on the cellulose surface in the form of a coating, which is covalently bonded to the surface of the nanocrystals. As polysiloxane continues to deposit around the cellulose nanofibrils, the cellulose chains exhibit a non-uniform widening feature [34].

**Figure 9:**
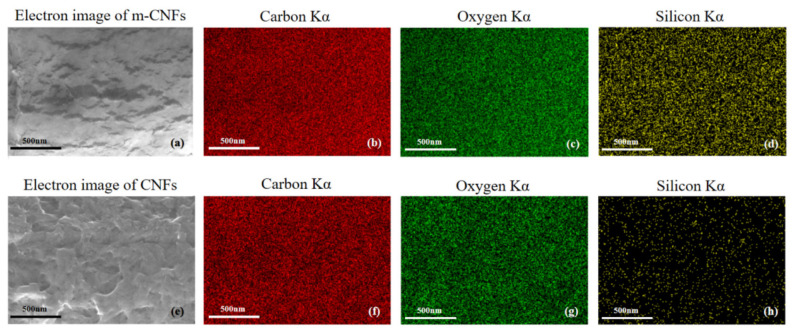
(**a**–**d**) SEM image and corresponding elemental mapping (C, O, Si) of m-CNFs; (**e**–**h**) SEM image and corresponding elemental mapping (C, O, Si) of CNFs.

The yellow regions in Figure 9d,h represent the EDS mapping of Si elements in m-CNFs and CNFs, respectively. The presence of silicon in the m-CNFs confirms that silane groups were successfully grafted onto the cellulose nanofibril surface, forming a continuous polysiloxane coating network.

### 3.2. Performance of JS Waterproof Coatings

#### 3.2.1. Tensile Strength and Bonding Strength

**Figure 10:**
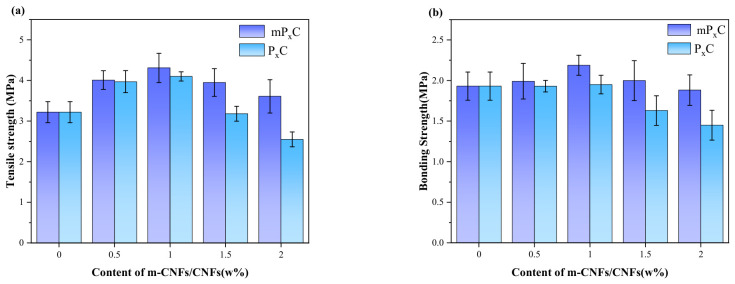
Mechanical properties of P_x_C and mP_x_C: (**a**) tensile strength; (**b**) bonding strength.

The tensile strength and bonding strength of coatings containing different contents of m-CNFs and CNFs are shown in Figure 10. These coatings were prepared using styrene–acrylate emulsions modified with different amounts of m-CNFs as the matrix. As shown in Figure 10a, with increasing m-CNF content, the tensile strength of the coatings first increased and then decreased, reaching a maximum value of 4.31 MPa, followed by a decline to 3.61 MPa. Notably, the tensile strength of the P_1.5_C coating reached 3.95 MPa, showing only a slight decrease of 8.3%. Compared with the P_0_C coating, the tensile strength of mP_1_C increased by 33.8%. This is consistent with the findings reported by Ashraf et al. [35]. The tensile strength of the P_1_C coating was 4.10 MPa. With an increase in the CNF content to 1.5 wt%, the strength decreased to 3.18 MPa (a reduction of 22.4%) and further dropped to 2.55 MPa for the P_2_C coating, corresponding to a total decrease of 37.8%.

Similarly, the P_x_C coatings containing CNFs exhibited the same trend at low CNF contents. This significant enhancement can be attributed to the three-dimensional reinforcing network structure formed by cellulose nanofibrils within the coating matrix, which effectively disperses external stress and reduces local stress concentration, thereby improving the tensile properties of the coating [36,37].

The results also indicate that the strength of coatings containing CNFs is generally lower than that of coatings containing m-CNFs. This can be attributed to the hydrophobic modification introduced by the silane reagent, which endows the surface of m-CNFs with both hydrophobic groups and residual hydrophilic hydroxyl groups, resulting in an amphiphilic structure. This amphiphilicity significantly improves the interfacial compatibility and dispersion stability of m-CNFs in the styrene–acrylate emulsion [38]. On the one hand, the silane groups partially replace hydroxyl groups, reducing intermolecular hydrogen bonding and thereby suppressing the aggregation of cellulose nanofibrils. On the other hand, the remaining hydrophilic groups maintain moderate interfacial activity, facilitating the uniform dispersion of m-CNFs within the coating [39]. This amphiphilic structure not only enhances the bridging effect of m-CNFs at the organic–inorganic interface but also improves the continuity of the coating microstructure, thereby strengthening the interfacial bonding and stress transfer efficiency between the reinforcing phase and the matrix [40,41].

In addition, the introduction of nanomaterials also contributes to the refinement of the internal structure. These materials can occupy pores and microcracks that develop during the curing stage, which reduces defects at the microscale and leads to an improvement in the tensile strength [42]. As the m-CNF content increases, the tensile strength shows a trend of initial increase followed by a decrease. At low contents, the excellent dispersion and favorable interfacial interaction of m-CNFs significantly improve the coating performance. At a relatively higher dosage (1.5 wt%), m-CNFs can still maintain relatively good dispersion even without the use of dispersants, allowing part of the reinforcement effect to be preserved [43]. However, excessive m-CNFs increase the viscosity of the coating, which adversely affects the rheological behavior during mixing with cement-based materials and ultimately leads to a loss of the self-leveling ability of the coating. Moreover, at a higher dosage (2 wt%), the aggregation of m-CNFs may occur within the coating, forming microstructural defects and resulting in a reduction in tensile strength [44].

As depicted in Figure 10b, the trend in the bonding strength of the JS waterproof coatings also shows an analogous pattern to that of the tensile strength, where an improvement followed by degradation is recorded as the m-CNF content increases. From the study, it was found that the mP_1_C waterproof coating possesses the highest bonding strength, reaching 2.12 MPa, an improvement of 9.8% over the P_0_C sample. The bonding strength of the P_1_C coating is higher than that of the Janus-type coating (2.33 MPa) reported by Li et al. [4], whereas its tensile strength is relatively lower.

The improved bonding strength of the coatings may be attributed to the incorporation of cellulose nanofibrils, as they provide an increased roughness at the interface, thus enhancing the interfacial contact area and improving the interfacial bond.

Nevertheless, when the m-CNF content becomes excessive, particle agglomeration may occur, which negatively affects the mechanical properties of the coating system. A similar phenomenon is observed in P_x_C coatings containing unmodified CNFs. At a dosage of 1.5 wt%, significant aggregation appears, resulting in a pronounced reduction with respect to the tensile and bonding strength.

#### 3.2.2. Contact Angle and Water Absorption

**Figure 11:**
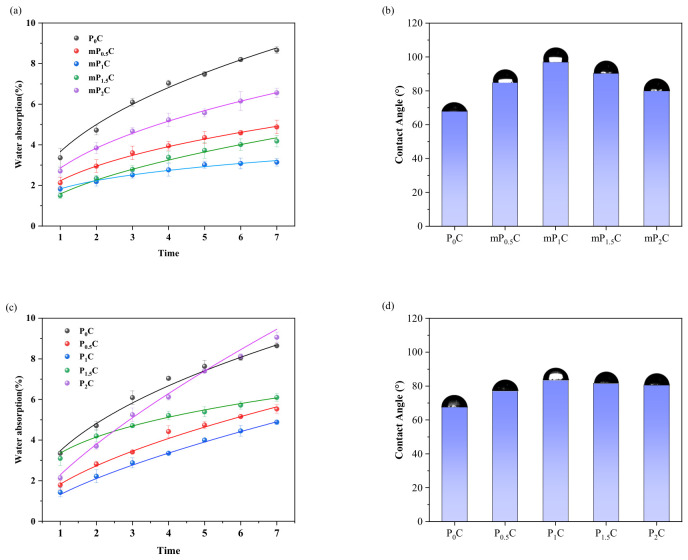
Water-repellent properties: (**a**) water absorption of mP_x_C; (**b**) contact angle of mP_x_C; (**c**) water absorption of P_x_C; (**d**) contact angle of P_x_C.

Figure 11 demonstrates the hydrophobic properties of JS waterproof coatings with varying dosages of modified cellulose nanofibrils and unmodified CNFs. As depicted in Figure 11a, the addition of an optimal dose of m-CNFs was observed to strongly improve the water resistance of the JS waterproof coatings. Of all the tested formulations, the P_1_C formulation exhibited the highest level of improvement in terms of water absorption, reaching only 1.9% after one day and 3.2% after seven days of immersion in water. Compared with the P_0_C reference sample, these results indicate a decrease in water absorption by 43.5% and 63.1%, respectively, for the P_1_C formulation after 1 day and 7 days.

This is primarily because the cellulose nanofibrils play a crucial role in the structure of the JS waterproof coating film. This is because cellulose nanofibrils have the capacity to fill micro- and nano-scale pores that are formed in the film after coating, as well as acting as effective nucleation sites for cement hydration products, thus enabling the formation of C-S-H gel, which in turn results in a denser structure. This results in a decrease in the porosity of the film, thus reducing the water absorption capacity [45].

The effect of the untreated CNFs on the water resistance is shown in Figure 11c. The coatings show a minor improvement in water resistance at relatively lower dosages of CNF, with the lowest water absorption recorded by the P_1_C coating after seven days of immersion at 5.0%. However, when the concentration of the CNF is increased to 2 wt%, the 7-day water absorption is significantly increased, reaching 9.1%, which is much higher than that of the P_0_C coating.

The surface hydrophilicity of the coatings was evaluated by determining the contact angles of the coatings with water. The contact angles of the coatings are shown in Figure 11b.

The contact angle of the P_0_C coating is relatively small at 68.3°, showing that the surface is not hydrophobic. The contact angles of the coatings increase with the addition of m-CNFs and then decrease with the addition of a high dose of m-CNFs. The mP_1_C coating shows the maximum contact angle of 97.1°, showing the best hydrophobicity of the coatings.

A similar tendency can also be seen in the P_x_C coatings, as depicted in Figure 11b. Among the coatings within this group, the largest contact angle is seen in the P_1_C coating at 85.2°, although this is still less than that seen in the mP_1_C coating.

The enhanced hydrophobicity associated with m-CNFs originates from the synergistic interaction between silane modification and nanoscale reinforcement. The introduction of silane functional groups decreases the surface energy of CNFs, while the incorporation of CNFs increases the roughness of the coating surface [46]. The combined effect of the reduced surface energy and increased surface roughness significantly strengthens the hydrophobic properties of the coating system.

#### 3.2.3. Water Permeability Resistance

**Figure 12:**
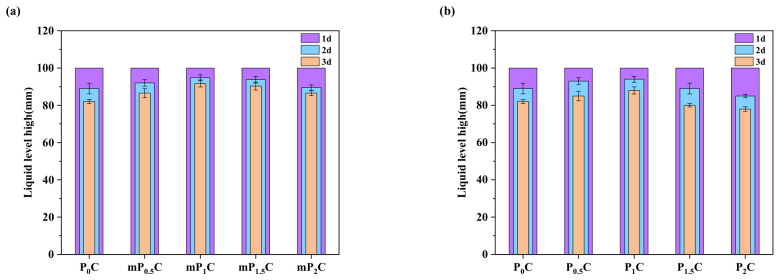
Liquid level diagrams of various coatings at different time periods: (**a**) mP_x_C; (**b**) P_x_C.

Figure 12a shows the water permeability resistance results of the mP_x_C coatings. The decrease in water level reflects the permeability resistance of the coatings. The results indicate that the water level of the P_0_C coating decreased by 18 mm within two days, indicating significant leakage. In comparison, the mP_0.5_C coating showed a smaller decrease, with the water level dropping by 13 mm within two days. The mP_1_C coating showed only a limited reduction in water level during the test period. The measured decreases were 3 mm after two days and 5 mm after three days, indicating a relatively stable water retention behavior. This slight change is mainly related to water uptake by the material and natural evaporation after curing. No clear signs of leakage were detected throughout the test. However, due to significant cracking in the coating, the mP_2_C sample exhibited a substantial water level decrease of 13 mm, indicating severe leakage.

Figure 12b presents the water permeability resistance results of the P_x_C coatings. The final water level shows a trend of first increasing and then decreasing with increasing CNF content. Among them, the P_1_C coating retained the highest water level among all samples during the test period. After two days, the water level decreased by 6 mm, and after three days, the reduction reached 12 mm. No obvious cracks were observed on its surface; however, the water level reduction was still larger than that of mP_1_C. This may be attributed to the aggregation of CNFs on the coating surface, which enables the coating to absorb more water compared with m-CNFs, resulting in a larger reduction in the water level.

Compared with mP_x_C coatings, the P_x_C coatings exhibited noticeable “spider-web” cracking at relatively low CNF contents. For example, the P_1.5_C coating showed water level decreases of 11 mm and 20 mm after two and three days, respectively, indicating severe leakage. This behavior can be attributed to the better dispersion of m-CNFs, which allows them to remain well dispersed even at relatively higher contents.

#### 3.2.4. Durability Test

Cellulose nanofibrils exhibits good intrinsic stability [47]. On this basis, silane modification can further improve the durability of the coatings [48,49].

**Figure 13:**
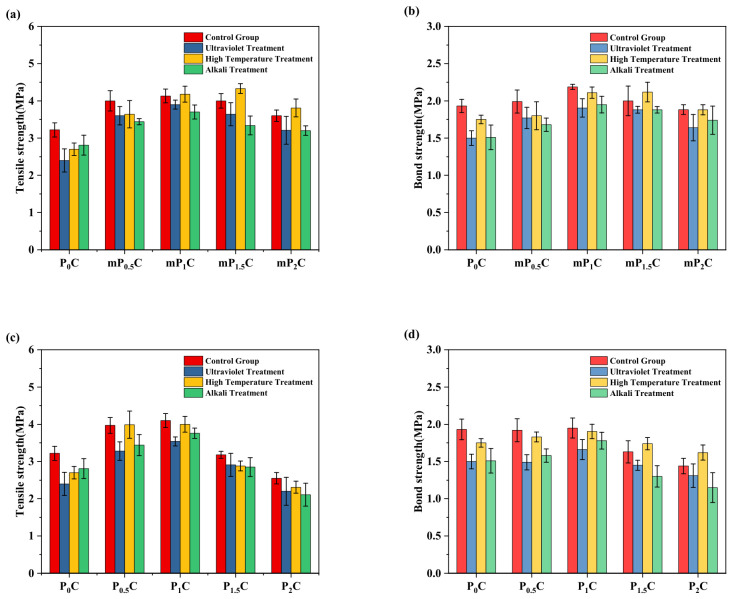
Comparison of mechanical performance of waterproof coatings under various aging conditions: (**a**) tensile strength of mP_x_C; (**b**) bonding strength of mP_x_C; (**c**) tensile strength of P_x_C; (**d**) bonding strength of P_x_C.

The experimental results are presented in Figure 13. The experimental groups containing m-CNFs exhibited significant enhancements in both bonding strength and tensile strength. After 720 h of ultraviolet irradiation, the tensile strength of all groups decreased to varying extents. Specifically, the tensile strength of mP_1_C decreased from 4.13 MPa to 3.91 MPa, corresponding to a reduction of only 5.3%, whereas that of P_1_C decreased from 4.10 MPa to 3.78 MPa (7.8% reduction), and the P_0_C coating declined more markedly from 3.22 MPa to 2.60 MPa, with a reduction of 20.5%. The bonding strength exhibited a similar trend.

These results demonstrate that the incorporation of CNFs and m-CNFs markedly enhances the UV-aging resistance of the coatings. It is well established that UV irradiation can induce powdering, cracking, and chemical bond scission in materials, ultimately leading to mechanical degradation. In addition to the intrinsic UV-shielding effect of cellulose nanofibrils, the polysiloxane network on their surface exhibits strong UV absorption and self-healing capabilities [50], thereby effectively mitigating UV-induced damage to the coatings.

After 7 days of high-temperature treatment, the tensile strength of the P_0_C, mP_0.5_C, and mP_1_C coatings decreased by 16.2%, 15.8%, and 3.2%, respectively. The results indicate that the introduction of m-CNFs enhances the thermal resistance of the coatings. Of note, the tensile strength of mP_1.5_C (4 to 4.33 MPa) and mP_2_C (3.60 to 3.81 MPa) coatings increased by 8.7% and 5.5%, respectively, after high-temperature treatment. A similar trend was also observed for the P_x_C coatings, and the increase was even more pronounced. This phenomenon can be attributed to the hydroxyl groups of cellulose nanofibrils, which retain a certain amount of free water through hydrogen bonding and the network structure. Under high-temperature conditions, the retained water molecules diffuse into the coating and react with cement particles through secondary hydration, producing an internal curing effect and thereby enhancing the coating strength [51].

Under alkaline immersion conditions, the alkaline solution can gradually infiltrate microdefects within the coating layer and attack the organic components.

This process may also cause the cleavage of chemical bonds existing in the polymer emulsion. This, in turn, may affect the bonding between the organic matrix and the inorganic materials. The results obtained from the experiments showed that after seven days of exposure to alkaline conditions, there was a reduction of 10–15% in both the tensile and bonding strength of all coating samples.

This is mainly due to the poor stability of the Si-O-C bond. In an aqueous alkaline solution, alkali-catalyzed hydrolysis of this bond results in the degradation of the siloxane network formed on the surface of the cellulose nanofibrils, resulting in reduced coating strength. The reaction mechanism of this alkali-catalyzed hydrolysis is based on the nucleophilic reaction of hydroxide ions with the silicon atom. However, the grafting of silane coupling agents onto cellulose nanofibrils increases the steric hindrance around the silicon atoms, which partially inhibits the hydrolysis of the siloxane network. As a result, the reduction in coating strength remains relatively limited.

#### 3.2.5. Adsorption–Desorption Curves and Pore Structure Analysis

**Figure 14:**
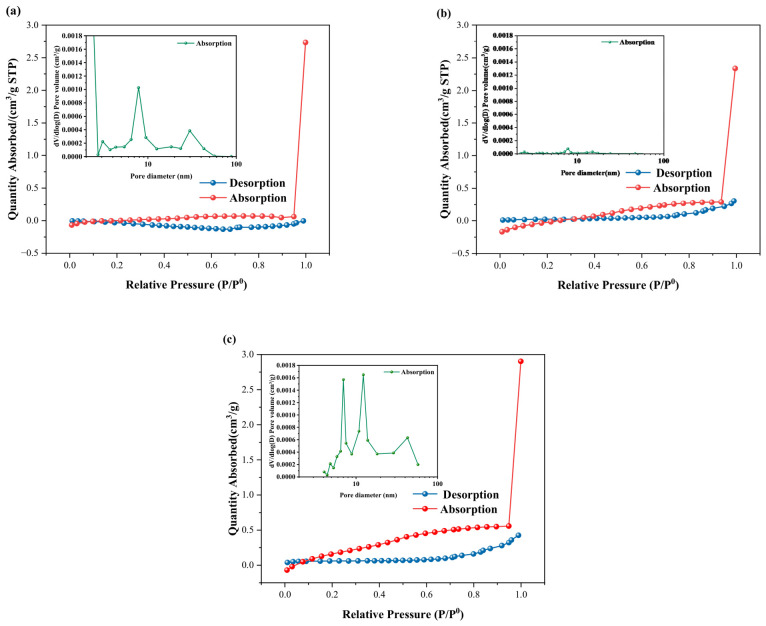
Adsorption–desorption isotherms and pore size distribution of coatings: (**a**) P_0_C; (**b**) mP_1_C; (**c**) P_1_C.

Figure 14 presents the nitrogen adsorption–desorption isotherms and the corresponding pore size distribution of the P_0_C, mP_1_C, and P_1_C coatings.

The adsorption–desorption curves of all coating samples display characteristic type IV isotherm features with evident hysteresis loops, suggesting that mesoporous structures are present within the coatings. The hysteresis loop originates from the capillary condensation phenomenon. During the increase in relative pressure (P/P0), nitrogen molecules first form multilayer adsorption films on the pore walls, followed by capillary condensation within mesopores. During the desorption process, the formation of a meniscus at the pore openings causes the evaporation pressure of condensed nitrogen to be lower than its condensation pressure. As a result, the adsorption and desorption paths do not coincide, leading to the formation of hysteresis loops.

The characteristics of the hysteresis loops, including their morphology, dimensions, as well as the closure pressure, are highly correlated with the pore structure of the material, i.e., the pore geometry, pore size distribution, as well as the surface properties of the material. In most cases, it has been noted that the adsorption–desorption curves of porous materials that have an almost uniform pore size distribution, along with a regular pore morphology, tend to approach an almost reversible equilibrium during the adsorption–desorption process. As a result, these types of materials tend to have relatively narrow hysteresis loops, or even negligible hysteresis, as observed in the isotherm curves. On the contrary, the wide hysteresis loops observed in the isotherm curves indicate the presence of a large number of irregularly shaped pores with a broad pore size distribution, including both irregular mesopores and macropores. These pore structures facilitate the rapid adsorption of nitrogen during the adsorption process. In contrast, the limited connectivity between pores, commonly referred to as the ink-bottle effect, significantly restricts the desorption of nitrogen, thereby leading to a pronounced hysteresis effect [52]. It is worth noting that the incomplete closure of the hysteresis loop observed in this work is not caused by experimental factors such as insufficient degassing or instrumental errors but is a normal intrinsic behavior of the material. The smooth and regular adsorption–desorption curves further exclude the interference of test conditions, which can be mainly attributed to the irreversible physical adsorption of N_2_ molecules in narrow pores and the unique pore structure characteristics of the sample. Combined with the pore size distribution curves, these results further confirm that the materials exhibit a hierarchical pore structure dominated by micropores and mesopores.

As shown in Figure 14a,c, distinct hysteresis loops are observed in the 0.1–1.0 range, while in Figure 14b, a hysteresis loop appears in the 0.4–1.0 range. This indicates that the mP_1_C coating possesses lower porosity, a more homogeneous distribution of pores and simpler pore structures.

Nitrogen adsorption isotherms were analyzed based on the Barrett–Joyner–Halenda (BJH) approach to obtain the pore size distribution in the 2–100 nm range. This analysis helps reveal the distribution and proportion of pores in the coatings. For the P_0_C coating, significant peaks appear at 7.5–8 nm and 28–32 nm, with maximum values at 7.8 nm and 29.8 nm, corresponding to 1.03 × 10^−3^ cm^3^/g and 3.84 × 10^−4^ cm^3^/g, respectively. These results indicate that the P_0_C coating contains a relatively large number of pores with a complex pore size distribution.

For the mP_1_C coating, peaks appear at 7.5–8 nm and 12–17 nm, with peak values of 7.95 × 10^−5^ cm^3^/g and 2.99 × 10^−5^ cm^3^/g, respectively. Compared with the P_0_C coating, the peak intensity of the mP_1_C coating is significantly reduced, indicating a much lower pore volume and improved microstructural compactness. This improvement is attributed to the introduction of m-CNFs, which enhances the interfacial bonding between the cement phase and the organic phase, thereby increasing the compactness of the material and improving its overall performance [4].

However, in the P_1_C coating, the peaks at 7.01 nm and 12.31 nm increase significantly, reaching 1.57 × 10^−3^ cm^3^/g and 1.65 × 10^−3^ cm^3^/g, respectively. In addition, another peak appears at 42.81 nm with a value of 6.33 × 10^−4^ cm^3^/g. This behavior can be attributed to the insufficient bridging effect of CNFs between the emulsion and fillers, which leads to an increase in micropores. Moreover, the aggregation of CNFs results in the formation of mesopores within the coating, reducing the compactness of the internal structure and introducing structural defects [53].

In conjunction with the pore structure characteristics discussed above, the water absorption behavior is closely associated with the pore size distribution and pore volume of the coatings. Compared with P_0_C, the significantly reduced pore peaks in mP_1_C indicate a lower pore volume and a more compact microstructure, which effectively limits water penetration and transport. In contrast, P_1_C exhibits increased pore volume and a broader pore size distribution, particularly with the emergence of larger mesopores, providing more accessible pathways for capillary water ingress. This difference is mainly attributed to the dispersion state of CNFs, where well-dispersed m-CNFs enhance interfacial bonding and densification, while CNF aggregation introduces additional pores and structural defects, thereby promoting water absorption.

#### 3.2.6. Microstructural Observation of Waterproof Coatings

This study further explores how modified cellulose nanofibrils affect the microstructural characteristics of JS waterproof coatings. As a nanoscale reinforcement material, the dispersion state of m-CNFs within the coating matrix plays a decisive role in determining waterproof performance. Poor dispersion or aggregation of nanofillers may introduce structural defects, which in turn reduce the protective capability of the coating system.

**Figure 15:**
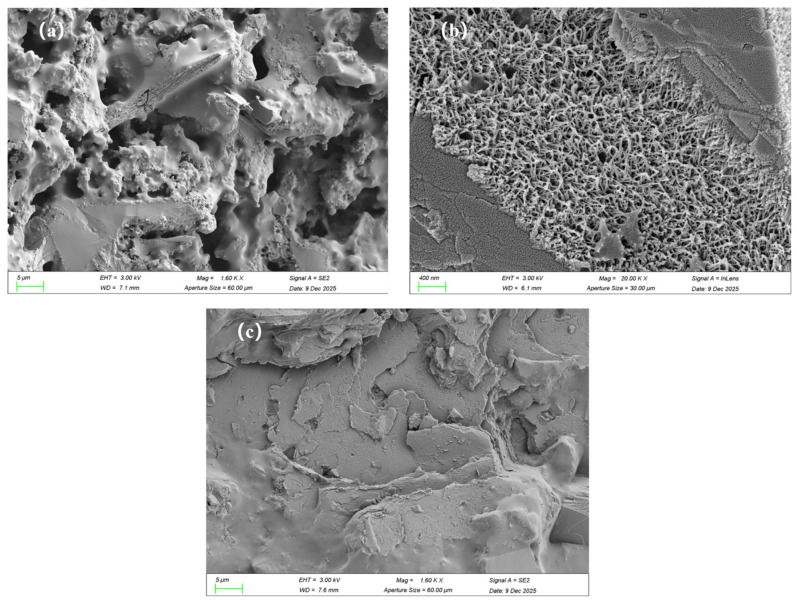
SEM images of JS waterproofing coatings: (**a**) P_0_C; (**b**) P_1_C; (**c**) mP_1_C.

The microstructure of the reference coating is presented in Figure 15a. It can be seen that after the curing process, there are many pores inside the P_0_C film. The pores are mainly caused by phase separation between the mineral filler and the polymer emulsion in the film-forming process. This suggests that the bonding between the polymer phase and the inorganic phase is relatively poor, which is recognized as a general drawback in traditional JS waterproof coatings.

The microstructural characteristics of the composite material consisting of the coating with the untreated CNFs are depicted in Figure 15b. It should be noted that the incorporation of CNFs into the composite material has dual effects on the structure of the composite material. On the one hand, in some areas, the structure of the composite material appears to be rather dense, indicating that the CNFs may be able to play a certain positive role in improving the interfacial interaction between the organic and inorganic phases and enhancing the compatibility of the phases. However, the existence of a large amount of hydroxyl groups on the surface of the CNF allows for the formation of hydrogen bonds, which may easily cause the aggregation of the CNF fibers and the formation of clusters during the course of the film formation process. These clusters may cause the formation of microcracks in the latex film, thereby damaging the structure of the composite material and, in turn, deteriorating the properties of the composite material.

In contrast, the dispersion of silane-modified m-CNFs is significantly improved. It can be seen from Figure 15c that the microstructure of the mP_1_C coating with m-CNFs is much denser, with fewer pores and defects. The silane groups replace some of the hydroxyl groups on the surface of cellulose nanofibrils, suppressing the hydrogen bonding between them and thus inhibiting aggregation. The amphiphilic nature of m-CNFs allows the emulsion to more efficiently entrap the cement particles, improving the adhesion between the two phases and resulting in a denser structure.

**Figure 16:**
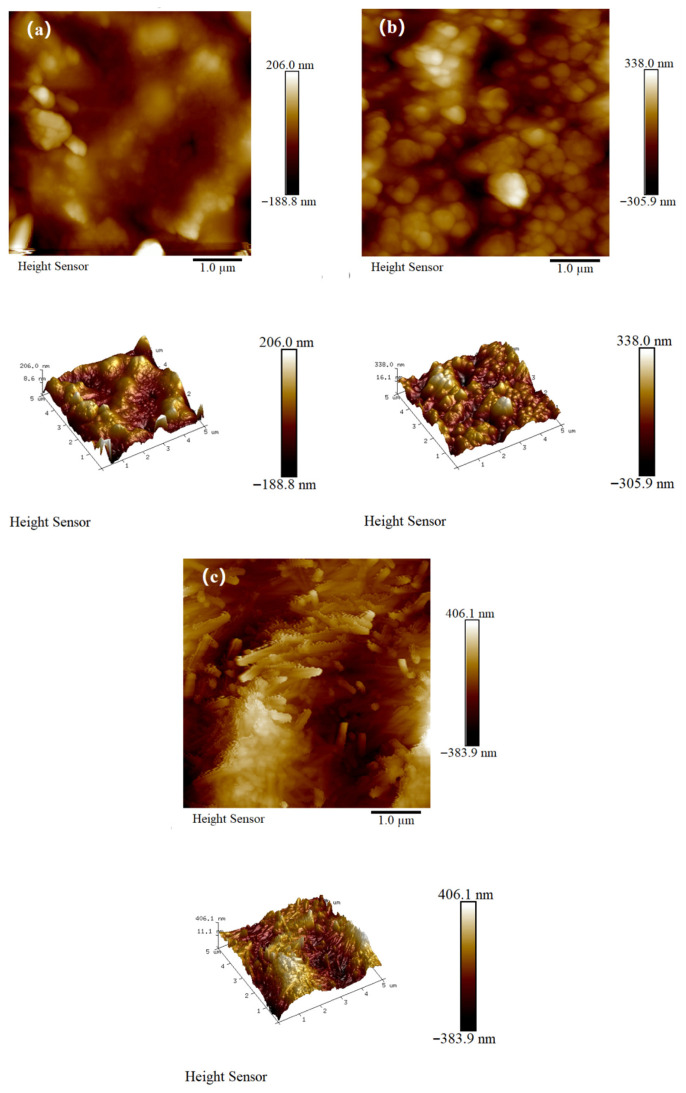
AFM images of JS waterproofing coatings: (**a**) P_0_C; (**b**) P_1_C; (**c**) mP_1_C.

To further shed light on the effect of m-CNFs on the surface properties of the formed coatings, atomic force microscopy was used for analyzing the surface topography, and the corresponding results are presented in Figure 16. As can be seen in Figure 16a, the surface morphology of the reference P_0_C coating is relatively smooth. On the contrary, the surface morphology of the P_1_C coating, which was formed in the presence of CNFs, has noticeable surface undulations, thus indicating increased surface roughness. It should be noted that, among all the analyzed samples, the surface irregularities are the most pronounced for the mP_1_C coating, thus indicating the highest surface roughness.

The quantitative surface roughness measurements also support these results. The arithmetic mean surface roughness (Ra) and the root mean square surface roughness (Rq) for the P_0_C coating were measured and found to be 54.2 nm and 42.5 nm, respectively. When CNFs are introduced into the coating, as in the P_1_C coating, the surface roughness is enhanced, as indicated by the increased values of Ra and Rq, which are 84.4 nm and 65.9 nm, respectively. The mP_1_C coating has the highest surface roughness, as indicated by the increased value of Ra and Rq, which are 123 nm and 102 nm, respectively, or twice those of the P_0_C coating.

The change in surface roughness can be largely explained by the addition of nanoscale fillers and the interactions with the polymer matrix. The nanoscale fillers have a tendency to accumulate and partially aggregate on the surface of the coating. This leads to a micro/nano-scale hierarchical structure on the surface of the coating. The depressions and protrusions of this rough surface structure are important in controlling the infiltration of water into the coating layer [54]. In addition, the addition of silane-modified cellulose nanofibrils, as well as the SiO_2_ particles resulting from the fillers, creates a specific surface structure similar to the Cassie–Baxter model [55]. This structure significantly increases the hydrophobicity of the coating.

#### 3.2.7. Waterproofing Mechanism of m-CNF-Modified JS Coatings

m-CNFs effectively bridge the SA emulsion and inorganic components (e.g., cement), integrating the structural integrity provided by the inorganic phase with the flexibility of the polymer matrix, thereby generating a synergistic enhancement in the overall performance [19]. This dual functionality not only improves the mechanical properties of the JS waterproof coating but also significantly enhances its resistance to water penetration.

**Figure 17:**
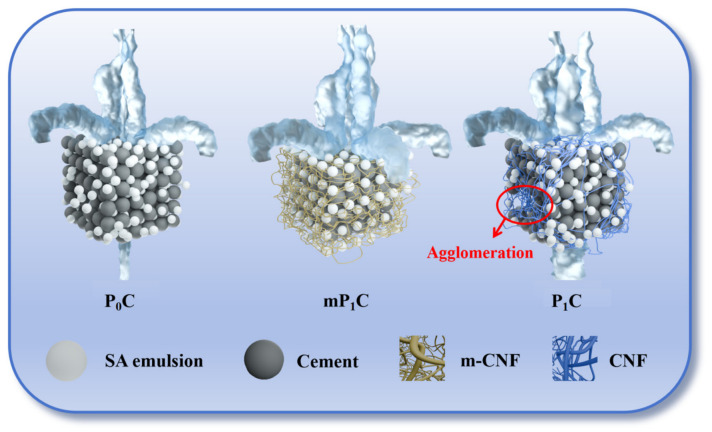
Types and anti-permeation mechanisms of JS waterproof coatings.

The waterproofing mechanism is schematically illustrated in Figure 17. For conventional SA coatings, interfacial defects may exist between the polymer film and the cement-based substrate, leading to structural discontinuities within the coating. These defects can serve as direct pathways for water ingress, allowing moisture to penetrate through the coating and reach the substrate, thereby causing leakage [56].

When 1 wt% CNFs are incorporated, the increased viscosity of the SA emulsion hinders the uniform dispersion of nanoparticles. Although CNFs can partially fill internal voids, their poor dispersion tendency leads to aggregation, which may form continuous water transport channels and compromise the waterproof integrity of the coating [35].

In contrast, coatings containing m-CNFs exhibit improved dispersion, primarily due to the silane modification, which enhances the interfacial adhesion between the organic polymer matrix and the inorganic components. This improved dispersion enables a more homogeneous integration of the rigid inorganic phase with the flexible polymer matrix. The small size of m-CNFs facilitates the effective sealing of micro-defects within the coating. In addition, the formation of a nano-scale rough structure enhances the compactness of the coating, improves mechanical strength, and contributes to enhanced hydrophobicity. Collectively, these features lead to a significant improvement in the overall waterproofing performance of the JS coating system [4,57].

From the perspective of practical application, the proposed coating system demonstrates promising economic, sustainable, and feasible characteristics. The incorporation of CNFs, derived from renewable and widely available biomass resources, contributes to the environmental sustainability of the material [58]. Meanwhile, the relatively low dosage required to achieve significant performance enhancement ensures that the additional material cost remains limited. In terms of feasibility, the preparation process is compatible with conventional coating fabrication methods and does not involve complex procedures or specialized equipment, indicating strong potential for large-scale implementation. These advantages suggest that the developed system is not only effective in performance but also viable for practical and sustainable engineering applications.

## 4. Conclusions

In this work, cellulose nanofibrils fibers were chemically modified through surface treatment with the silane coupling agent vinyltriethoxysilane. The obtained modified cellulose nanofibrils were characterized using Fourier transform infrared spectroscopy, scanning electron microscopy, and energy-dispersive spectroscopy. The modified nanofibers were subsequently incorporated into a styrene–acrylate emulsion and combined with cement and inert fillers to fabricate JS waterproof coatings. The performance and underlying mechanisms of the resulting coatings were systematically investigated through mechanical tests, water resistance measurements, permeability evaluation, durability assessment, BET, and AFM analysis. The major findings of this study are summarized below.

(1) When the m-CNF dosage reached 1 wt%, the JS waterproof coating achieved the best mechanical performance. Under this condition, the tensile strength and bonding strength reached 4.31 MPa and 2.12 MPa, respectively. Compared with the reference P_0_C coating, these values correspond to increases of 33.8% in tensile strength and 9.8% in bonding strength. The improvement mainly arises from the three-dimensional reinforcing network formed by cellulose nanofibrils within the coating matrix. This network structure effectively redistributes external stress, reduces internal porosity, and mitigates localized stress concentration, thereby enhancing the overall mechanical performance of the coating.

(2) The coating incorporating 1 wt% m-CNFs (mP_1_C) exhibited a water absorption of 3.2% after seven days of immersion, corresponding to a 72.9% decrease compared with the P_0_C sample. Meanwhile, the water contact angle increased to 97.1°, demonstrating a pronounced improvement in hydrophobic performance. During the permeability assessment, the coating surface remained intact without observable cracking after seven days, and the water level in the apparatus showed negligible decline. These findings indicate that the introduction of m-CNFs effectively suppresses pore generation and enhances the compactness of the coating microstructure.

Furthermore, the surface roughness increased significantly, with Ra and Rq rising from 54.2 nm and 42.5 nm to 123 nm and 102 nm, respectively. This enhancement in waterproofing behavior can be attributed to the synergistic effect of the increased microscale roughness induced by the nano-fillers and the reduced surface energy resulting from silane modification. Such a combination is likely to induce a Cassie–Baxter-like wetting state, thereby providing a plausible mechanistic explanation for the improved water repellency.

(3) The incorporation of m-CNFs also significantly enhanced the durability of the JS waterproof coatings. After exposure to high-temperature conditions, the tensile strength of the P_0_C and mP_1_C coatings decreased by 16.2% and 3.2%, respectively, whereas the tensile strength of mP_1.5_C and mP_2_C coatings increased by 8.7% and 5.5%. This phenomenon can be attributed to the gradual release of absorbed water from cellulose nanofibrils at elevated temperatures, which promotes secondary hydration reactions with cement. Following ultraviolet irradiation, the tensile strength of the mP_1_C coating decreased by only 3.2%, compared with reductions of 5.4% for P_1_C and 19.2% for P_0_C. These results demonstrate that the modified cellulose nanofibrils significantly improves the resistance of the coating to UV degradation. The enhanced UV stability is mainly associated with the inherent stability of cellulose nanofibrils and the ultraviolet shielding effect provided by the siloxane network generated during silane modification.

Nevertheless, several limitations of the present study should be acknowledged. Although the surface modification of CNFs can effectively mitigate their tendency to aggregate, our results indicate that aggregation may still occur when the CNF content exceeds 1.5 wt%, which could adversely affect the overall performance of the coating. In addition, it should be noted that the developed coating is not designed to provide thermal insulation. The performance evaluation in this study is therefore limited to indoor application scenarios, and the behavior of the coating under more extreme environmental conditions remains to be further investigated.

## Figures and Tables

**Table 1:** Chemical composition of cement (%).

Composition	CaO	Fe_2_O_3_	Al_2_O_3_	MgO	SiO_2_	SO_3_	NaO_2_	LOI
Content/wt%	54.37	5.61	5.85	5.22	21.15	2.60	1.42	1.22

**Table 2:** Mineral composition of cement (%).

Clinker Mineral	C_3_S	C_2_S	C_3_A	C_4_AF
Content/wt%	59.61	14.61	7.12	10.34

**Table 3:** Physical indicators of reference cement.

Density (g/cm^3^)	Specific Surface Area (m^2^/kg)	Fineness (80 µm)	Setting Time (min)		Flexural Strength (MPa)		Compressive Strength (MPa)	
			Initial	Final	3d	28d	3d	28d
3.16	352	0.006	139	189	6.3	13.1	27.5	42.9

**Table 4:** Key physicochemical properties of the SA emulsion.

Type	Particle Size (nm)	Solid Content (%)	Glassed Transition Temperature	Surface Charge Density (µeq/g)
SA	250	50	−5	−190

**Table 5:** Information on the reagents used in this study.

Reagent	Purity	Source of Material
Ethyl alcohol	98% (RG)	Adamas
Acetic acid	98% (RG)	
Acetone	98% (RG)	
Vinyltriethoxysilane (VTES)	98% (RG)	

**Table 6:** Mix proportions of JS waterproof coatings.

Group	Type	Polymer (g)	Cement (g)	Quartz Sand (g)	Heavy Calcium Carbonate Oxide (g)
P_0_C	SA	22.2	20	12	8
P_0.5_C	CNF_0.5_SA				
P_1_C	CNF_1_SA				
P_1.5_C	CNF_1.5_SA				
P_2_C	CNF_2_SA				
mP_0.5_C	m-CNF_0.5_SA				
mP_1_C	m-CNF_1_SA				
mP_1.5_C	m-CNF_1.5_SA				
mP_2_C	m-CNF_2_SA				

## Data Availability

The original contributions presented in this study are included in the article. Further inquiries can be directed to the corresponding author.

## Abbreviations

The following abbreviations are used in this manuscript:

JS	Polymer–cement
CNFs	Cellulose nanofibrils
VTES	Vinyltriethoxysilane
m-CNFs	Modified cellulose nanofibrils
FTIR	Fourier transform infrared spectroscopy
SEM	Scanning electron microscopy
EDS	Energy-dispersive X-ray spectroscopy
BET	Brunauer–Emmett–Teller
SA	Styrene–acrylate
CNCs	Cellulose nanocrystals
UV	Ultraviolet
BJH	Barrett–Joyner–Halenda
AFM	Atomic force microscopy

## References

[1] Nevalainen A., Partanen P., Jääskeläinen E., Hyvärinen E., Koskinen O., Meklin T., Vahteristo M., Koivisto J., Husman T. (1998). Prevalence of Moisture Problems in Finnish Houses. Indoor Air.

[2] Zhang Y., Li H., Jin D. (2013). The Leaking Protection Technology of Structural Deformation Joint after Cracking. Appl. Mech. Mater..

[3] Yan F.Y., Zhao C.Y. (2012). Mechanism of film formation and application of polymer cement waterproof coating. New Build. Mater..

[4] Li X., Wang Q., Wang J., Zeng F., Xu K. (2025). Preparation of Janus-type styrene-acrylic emulsions and performance in polymer cement waterproof coatings. Constr. Build. Mater..

[5] Fu W., Wang L., Huang J.Z. (2011). Polymer cement waterproof coating and its properties. Adv. Mater. Res..

[6] Qian X., Zhu A., Ji L. (2013). Organosilicone modified styrene-acrylic latex: Preparation and application. Polym. Bull..

[7] Patil S.R., Parekh N.C., More A.P. (2025). An overview of styrene acrylic emulsions: Synthesis, properties, and applications. Polym. Bull..

[8] Yang L.X., Gu X.J., Liu J.X., Wu L., Qin Y. (2024). Functionalized nanomaterials-based electrochemiluminescent biosensors and their application in cancer biomarkers detection. Talanta.

[9] Sun W., Tang E., Zhao L., Yuan M., Liu S., Xing X., Liu X. (2023). The waterborne epoxy composite coatings with modified graphene oxide nanosheet supported zinc ion and its self-healing anticorrosion properties. Prog. Org. Coat..

[10] Liu L., Zhao M., Pei X., Liu S., Luo S., Yan M., Shao R., Sun Y., Xu W., Xu Z. (2023). Improving corrosion resistance of epoxy coating by optimizing the stress distribution and dispersion of SiO_2_ filler. Prog. Org. Coat..

[11] Ma H., Huang H., Zhou M., Graham J., Smith X., Sheng Y., Chen L., Zhang X., Zhang E., Shchukina D. (2021). Superior anti-corrosion and self-healing bifunctional polymer composite coatings with polydopamine modified mesoporous silica/graphene oxide. J. Mater. Sci. Technol..

[12] Quaresimin M., Schulte K., Zappalorto M., Chandrasekaran S. (2016). Toughening mechanisms in polymer nanocomposites: From experiments to modelling. Compos. Sci. Technol..

[13] Li Z.Y., Shen J.H., Ma H.Q., Liu F.Y., Yu W.W., Shangguan Y.G., Zheng Q. (2025). Effect of partially crosslinked nanoparticle cluster networks in dispersed rubber phase on the toughness and stiffness of polypropylene composites. Compos. Part. B Eng..

[14] Hajibabazadeh S., Razavi Aghjeh M.K., Mehrabi Mazidi M. (2020). Stiffness-toughness balance in PP/EPDM/SiO_2_ ternary blend-nanocomposites: The role of microstructural evolution. J. Compos. Mater..

[15] Ibrahim A., Klopocinska A., Horvat K., Abdel Hamid Z. (2021). Graphene-based nanocomposites: Synthesis, mechanical properties, and characterizations. Polymers.

[16] Jawaid M., Abdul Khalil H.P.S. (2011). Cellulosic/synthetic fibre reinforced polymer hybrid composites: A review. Carbohydr. Polym..

[17] Shankar A., Abdul Malik A.K., Narayan R., Chakrabarty A. (2023). Emulsion polymerized styrene acrylic/cellulose nanofibrils composite coating to improve the strength and hydrophobicity of kraft paper. Prog. Org. Coat..

[18] Hu F., Zheng Z., Guo A., Sun Z., Yu Z., Yao C., Du Y. (2025). Mechanical properties and microstructure of cellulose nanocrystal modified cement pastes subject to chloride erosion. J. Build. Eng..

[19] Zhu K., Liang Y., Yuan J., Yu H., Jiang L., Wang J., Zhang J., Zhang J., Song D., Xia L. (2025). Flexible cement fibers with high toughness and water-activated setting behavior for construction. Nat. Commun..

[20] Eyley S., Thielemans W. (2014). Surface modification of cellulose nanocrystals. Nanoscale.

[21] Habibi Y. (2014). Key advances in the chemical modification of cellulose nanofibrilss. Chem. Soc. Rev..

[22] Castro Cabrera I., Berlioz S., Fahs A., Louarn G., Carriere P. (2020). Chemical functionalization of nano fibrillated cellulose by glycidyl silane coupling agents: A grafted silane network characterization study. Int. J. Biol. Macromol..

[23] Dhali K., Daver F., Cass P., Adhikari B. (2022). Surface modification of the cellulose nanocrystals through vinyl silane grafting. Int. J. Biol. Macromol..

[24] (2011). Test Methods for Water Requirement of Normal Consistency, Setting Time and Soundness of Portland Cement.

[25] (2021). Test Method of Cement Mortar Strength (ISO Method).

[26] Frank B.P., Durkin D.P., Caudill E.R., Zhu L., White D.H., Curry M.L., Pedersen J.A., Fairbrother D.H. (2018). Impact of silanization on the structure, dispersion properties, and biodegradability of cellulose nanofibrils as a nanocomposite filler. ACS Appl. Nano Mater..

[27] Rajan S.T.K., Nagarajan K.J., Balasubramani V., Sathickbasha K., Sanjay M.R., Siengchin S., Balaji A.N. (2023). Investigation of mechanical and thermo-mechanical characteristics of silane-treated cellulose nanofibers from agricultural waste reinforced epoxy adhesive composites. Int. J. Adhes. Adhes..

[28] Wang Q., Chen X., Zeng S., Chen P., Xu Y., Nie W., Xia R., Zhou Y. (2023). In-situ polycondensate-coated cellulose nanofiber heterostructure for polylactic acid-based composites with superior mechanical and thermal properties. Int. J. Biol. Macromol..

[29] Xia L., Zeng J., Xiao Y., Gong J., Chen Y. (2024). Surface-grafting modification of attapulgite nanorods with polysiloxane coupling agents for highly-efficient mechanical and triboelectric performance enhancement of silicone rubbers. Compos. Part. B Eng..

[30] (2009). Polymer-Modified Cementitious Waterproofing Coatings.

[31] (2008). Test Methods for Building Waterproofing Coatings.

[32] Yu C.C., Chiang K.Y., Okuno M., Seki T., Ohto T., Yu X., Korepanov V., Hamaguchi H., Bonn M., Hunger J. (2020). Vibrational couplings and energy transfer pathways of water’s bending mode. Nat. Commun..

[33] Ye Q., Meng X. (2022). Highly efficient authentication of edible oils by FTIR spectroscopy coupled with chemometrics. Food Chem..

[34] Zhang Z., Tingaut P., Rentsch D., Zimmermann T., Sebe G. (2015). Controlled silylation of Cellulose Nanofibrils in water: Reinforcement of a model polydimethylsiloxane network. ChemSusChem.

[35] Ashraf M.A., Peng W., Zare Y., Rhee K.Y. (2018). Effects of size and aggregation/agglomeration of nanoparticles on the interfacial/interphase properties and tensile strength of polymer nanocomposites. Nanoscale Res. Lett..

[36] Hu K., Wang Y.Y., Wang H.Y., Zhao Z., Liu K., Huang L., Chen L. (2023). Preparation of high-strength and low-temperature-resistant cellulose nanofibrils/polyvinyl alcohol conductive composite hydrogel and its application in flexible sensing. Acta Mater. Compos. Sin..

[37] Xu J., Deng X., Dong Y., Zhou Z., Zhang Y., Yu J., Cai J., Zhang Y. (2020). High-strength, transparent and superhydrophobic cellulose nanofibrils/nanochitin membranes fabricated via crosslinking of nanofibers and coating F-SiO_2_ suspensions. Carbohydr. Polym..

[38] Guan J., Lan J., Huang T., Xiao J., Wang J., Wei F., Zhang X.H. (2025). Enhanced anticorrosion and wear resistance of epoxy coatings via surface amphiphilic functionalization of silica fillers. Colloids Surf. A.

[39] Mekonnen T.H., Haile T., Ly M. (2021). Hydrophobic functionalization of cellulose nanocrystals for enhanced corrosion resistance of polyurethane nanocomposite coatings. Appl. Surf. Sci..

[40] Shrestha S., Chowdhury R.A., Toomey M.D., Betancourt D., Montes F., Youngblood J.P. (2019). Surface hydrophobization of TEMPO-oxidized cellulose nanofibrils using aqueous modification process and its effect on properties of epoxy nanocomposites. Cellulose.

[41] Yamato K., Yoshida Y., Kumamoto Y., Isogai A. (2021). Surface modification of TEMPO-oxidized cellulose nanofibers and properties of their acrylate and epoxy resin composite films. Cellulose.

[42] Wang T., Xu J., Zhu C., Ren W. (2019). Comparative study on the effects of various modified admixtures on the mechanical properties of styrene-acrylic emulsion-based cement composite materials. Materials.

[43] Yokota S., Tagawa S., Kondo T. (2021). Facile surface modification of amphiphilic cellulose nanofibrils prepared by aqueous counter collision. Carbohydr. Polym..

[44] Li G., Hu W., Cui H., Zhou J. (2019). Long-term effectiveness of carbonation resistance of concrete treated with nano-SiO_2_ modified polymer coatings. Constr. Build. Mater..

[45] Zaoui A., Ben Rejeb Z., Kwon O.S., Park C.B. (2025). Fine-tuning the microstructure of concrete composite: Impact of surface-coated cellulose nanofibrils on strength and variation reduction. Constr. Build. Mater..

[46] Shin E.A., Kim G.H., Jung J., Lee S.B., Lee C.K. (2021). Addition of cellulose nanofibers to control surface roughness for hydrophobic ceramic coatings. J. Nanosci. Nanotechnol..

[47] Manimaran M., Norizan M.N., Kassim M.H.M., Adam M.R., Norrrahim M.N.F., Knight V.F. (2024). Critical assessment of the thermal stability and degradation of chemically functionalized cellulose nanofibrils-based polymer nanocomposites. Nanotechnol. Rev..

[48] Li Y., Li Z., He Y., Wang K., Li D. (2022). Facile synthesis of atomic oxygen-resistant methyl silicone rubber-coated Kapton film for photovoltaic solar array blanket in low Earth orbit. J. Coat. Technol. Res..

[49] Shi S., Zhang D., Bi L., Ding R., Ren W., Tang X., He Y. (2024). Enhancement of thermal conductivity and insulation of silicone thermal interface material through surface modification and synergistic effects of nanofillers. Diamond Relat. Mater..

[50] Wei D., Liang S., Lv S., Zuo J., Liu L., Zhang S. (2024). Dodecyl sulphide silane-modified SiO_2_/lignin-based carbon nanoparticles superhydrophobic and UV-resistant composite coatings. J. Environ. Chem. Eng..

[51] Barnat-Hunek D., Szymańska-Chargot M., Jarosz-Hadam M., Łagód G. (2019). Effect of cellulose nanofibrils and nanocrystals on physical properties of concrete. Constr. Build. Mater..

[52] Kai D., Guanhua N., Yuhang X., Meng X., Wang H., Shang L., Qian S., Yunfei L. (2020). Effect of nano-SiO_2_/styrene-acrylic emulsion on compactness and strength of mine drilling seal materials. Powder Technol..

[53] Duan S., Gao W., Tang X., Liu H., Li J., Cao D., Zeng J., Wang B., Xu J., Chen K. (2025). Structural properties of redispersed cellulose nanofibrils analyzed via solute exclusion technique. Cellulose.

[54] Zhang C., Wu Y., Zhang Y., Sun Y. (2013). Research status of nanometer silicon dioxide’s modification of coatings. For. Mach. Woodwork. Equip..

[55] Xu R., Ye H., Wu M., Wu Q., Yang J., Liu J., Zhang J. (2024). Preparation of transparent superamphiphobic coating by rationally designed rough structure. J. Coat. Technol. Res..

[56] Scrivener K.L., John V.M., Gartner E.M. (2018). Eco-efficient cements: Potential economically viable solutions for a low-CO_2_ cement-based materials industry. Cem. Concr. Res..

[57] Yang X., Cranston E.D. (2014). Chemically cross-linked cellulose nanocrystal aerogels with enhanced mechanical properties. Chem. Mater..

[58] Klemm D., Cranston E.D., Fischer D., Gama M., Kedzior S.A., Kralisch D., Kramer F., Kondo T., Lindström T., Nietzsche S. (2018). Nanocellulose as a natural source for groundbreaking applications in materials science: Today’s state. Mater. Today.

